# Characteristics of adverse reactions to kratom and implementation of harm reduction strategies in a sample of ethnobotanical tea bar patrons in Colorado

**DOI:** 10.1186/s12954-025-01382-x

**Published:** 2026-03-02

**Authors:** Cianna J. Piercey, Joseph Cameron, Riley Ahern, Isabella Packwood, Carter Bruning, Devin Henry, Jesse Ruehrmund, Katelyn Weldon, Kirsten E. Smith, Hollis C. Karoly

**Affiliations:** 1 Department of Psychology, Colorado State University, Fort Collins, CO 80521 USA; 2 School of Social Work, Columbia University, New York, NY 10027 USA; 3 Department of Psychiatry and Behavioral Science, Johns Hopkins School of Medicine, Baltimore, MD 21205 USA; 4 Department of Psychiatry, University of Colorado Anschutz Medical Campus, Aurora, CO 80045 USA; 5 Center for Alcohol and Addiction Studies, Brown University School of Public Health, RI 02903 Providence, USA

**Keywords:** Kratom, Mitragyna speciosa, Ethnobotanical tea bars, Mixed methods, Field survey, Adverse effects, Harm Reduction

## Abstract

**Background:**

Kratom is a psychoactive botanical that is increasingly popular in the United States. While potential risks of kratom use have been identified, research on harm reduction strategies and contextual factors contributing to adverse reactions is limited. Given that kratom is federally unregulated at present, and a variety of kratom products are widely available on the commercial market, more data are needed to inform harm reduction efforts and public health messaging.

**Methods:**

102 participants (mean age = 22.34 years, 39.2% women) were recruited from ethnobotanical tea bars in Northern Colorado to complete a survey on kratom use, which included questions pertaining to adverse experiences.

**Results:**

Most participants (75.5%) reported experiencing an adverse reaction to kratom at least once in the past, with a wide range of kratom doses reportedly consumed during these experiences. Adverse reactions were most commonly reported to occur in the context of consuming kratom on an empty stomach, consuming alongside certain foods, lack of hydration, and combining kratom with other substances. Participants employed a variety of strategies in attempt to mitigate adverse reactions, such as stopping or pacing their use of kratom and other substances, modulating hydration and food intake, and engaging in rest and recovery behaviors.

**Conclusions:**

Adverse reactions to kratom may be associated with certain contextual factors. Several techniques are reportedly used to mitigate adverse reactions, but the efficacy of these techniques is unknown. More data are needed to understand the causes and consequences of adverse reactions to kratom, and to better understand potential harm reduction strategies for reducing adverse experiences. More research could also shed light on the extent to which kratom serving size and various product formulations (e.g. whole leaf vs. extracts) are predictive of adverse effects.

## Introduction

Kratom (Mitragyna sadenosinergicpeciosa Korth) is a psychoactive botanical native to Southeast Asia, the leaves of which have been shown preclinically to have opioidergic, , adrenergic, serotonergic, and dopaminergic activity [1–6]. While kratom has been used for medicinal purposes in Southeast Asia for centuries, it has more recently gained popularity in the United States over the past two decades [7]. 

Kratom consumers report multiple use motivations, such as increased focus, pain management, and alleviation of anxiety, depression, and substance use disorder symptoms [9–11]. Risks of kratom have also been identified, such as acute adverse reactions (e.g., gastrointestinal upset, dizziness) [12, 13] and the development of physical dependence or use disorder consistent with substance use disorder criteria outlined in the Diagnostic and Statistical Manual of Mental Disorders (DSM-5) [14–19]. While rare, toxicity and overdose fatalities have also been reported, typically involving coingestants [20, 21].

Despite potential risks, research on characteristics of acute adverse reactions to kratom is limited, such as dose consumed and other contextual factors [22]. Additionally, while harm reduction strategies have been studied across a range of substances (e.g., alcohol, cannabis, opioids, stimulants, psychedelics) [23–32, 32–36], data on kratom harm reduction strategies is currently lacking, particularly strategies used in attempt to mitigate acute adverse effects [37].

### Current study

We sought to document characteristics of kratom-related adverse reactions, and the strategies employed in attempt to reduce such harms, through asking participants open-ended questions about prior experiences of acute adverse reactions to kratom. Data presented in this report was collected as part of a pilot study on kratom use among ethnobotanical tea bar patrons in Colorado [38], a population which is underrepresented in the kratom literature at present (according to an informal search we conducted in the spring of 2024, we estimate that there are at least 300 ethnobotanical tea bars in the US that serve kratom products across at least 28 US states, yet other than our pilot work, there are no published data to our knowledge collected from individuals who patron these establishments). In our prior work [38], we provided a broad-level overview of kratom use at ethnobotanical tea bars which included data on use patterns, subjective effects, perceived benefits, and the types of adverse reactions that participants reported experiencing (e.g., nausea, drowsiness). In the current manuscript, we explore adverse reactions in more detail, including dose consumed, contextual factors contributing to adverse reactions, duration of adverse effects, employment of harm reduction strategies, and whether participants sought medical care following an adverse reaction.

## Methods

### Participants

Participants (Table 1) were 102 bar patrons aged 18–51 (mean age = 22.34, SD = 5.45) recruited from ethnobotanical tea bars in Northern Colorado (data were collected in the fall of 2023 and spring of 2024, prior to the introduction of regulation in Colorado restricting the sale of kratom to individuals 21 years or older). Individuals who did not report past-year kratom use (*N* = 4) were excluded from analysis.

**Table 1:** Participant characteristics

Characteristics	*N*	%
Gender		
Gender fluid	3	3.1
Gender queer	1	1.0
Man	53	54.6
Woman	38	39.2
Non-binary	2	2.1
Transgender		
Yes	3	3.1
No	92	94.8
Prefer not to answer	2	2.1
Ethnicity		
Arab, Middle Eastern, or North African	8	8.2
Asian or Asian American	10	10.2
Black or African American	5	5.1
Hispanic or Latino	19	19.4
Native American or Alaska Native	1	1.0
Native Hawaiian or Other Pacific Islander	2	2.0
White or European American	73	74.5
Not listed	5	5.1
Prefer not to answer	2	2.0
Race		
Asian	5	5.1
Black	2	2.0
Indigenous, Aboriginal, or First Nations	1	1.0
Latino or Hispanic	12	12.2
Middle Eastern	0	0.0
White	85	86.7
Not listed	0	0.0
Prefer not to answer	1	1.0
Sexual Orientation		
Straight or heterosexual	68	69.4
Lesbian	6	6.1
Gay	2	2.0
Bisexual	12	12.2
Pansexual	5	5.1
Sexually fluid	2	2.0
Queer	4	4.1
Demisexual	1	1.0
Asexual	1	1.0
Aromantic	0	0.0
Questioning	1	1.0
I use a different term	0	0.0
Prefer not to answer	0	0.0
Education		
Less than high school	5	5.1
High school diploma or GED	27	27.6
Some college	45	45.9
Associates degree or technical certification	10	10.2
Bachelor’s degree	8	8.2
Master’s degree	3	3.1
Doctoral degree	0	0.0
Household Income		
$0-$9,999/yr	24	24.5
$10,000-$19,999/yr	9	9.2
$20,000-$29,999/yr	7	7.1
$30,000-$39,999/yr	15	15.3
$40,000–49,999/yr	8	8.2
$50,000-$59,999/yr	7	7.1
Over $60,000/yr	18	18.4
Prefer not to answer	10	10.2

Note. M, mean. SD, standard deviation

### Procedure

Street-intercept methods were used to conduct in-person field surveys on kratom use at ethnobotanical tea bars in northern Colorado. Participants were asked whether they had ever experienced any negative or unwanted effects of kratom within a single session of use (e.g., a night out). If participants responded “yes” to this question, they were prompted to describe the effects they experienced [38] and answer additional questions on characteristics of the adverse reaction, detailed below. If participants had experienced an adverse reaction on more than one occasion, they were instructed to answer questions in terms of their most recent adverse experience. Participants were compensated $10 for the completion of the survey. The study was approved by the Institutional Review Board at Colorado State University.

### Measures

All items were open-ended text-response questions, except for the question about seeking medical attention, which was provided in a binary yes/no format.

***Kratom Dose.*** “What dose of kratom did you consume when you experienced negative or unwanted effects?”

***Contextual Factors.*** “Aside from the dose you consumed, were there any other factors that may have contributed to experiencing unwanted or negative effects?”

***Duration of Effects.*** “How long did it take for the negative or unwanted effects to wear off?”

***Harm Reduction Strategies.*** “Is there anything you did to lessen the negative or unwanted effects you were experiencing?”

***Medical Attention.*** “Did you seek out any medical attention after experiencing negative or unwanted effects?”

### Analysis

Kratom dose, duration of adverse effects, and incidence of seeking medical attention items are reported descriptively. Thematic analysis was used to generate relevant themes pertaining to contextual factors and harm reduction strategies [39]. Specifically, the first (CJP) and last author (HCK) engaged in familiarization with the data and created codes for each question using an inductive coding approach. Once CJP and HCK established an agreed upon codebook, they independently coded each participant response. After coding each question, they identified and resolved any discrepancies in their coding until they achieved 100% agreement. CJP and HCK then generated and refined themes for each question (Table 2).

## Results

Most participants (*N* = 74, 75.5%) endorsed at least one prior experience of negative or unwanted acute kratom effects, while 21.4% of participants denied having ever experienced an adverse reaction; 3.1% did not provide a response. Kratom dose consumed during participants’ most recent kratom-related adverse experience varied, with one participant reporting as little as one cup of tea and another participant reporting as many as 17 kratom “slapshots” (i.e., concentrated extract products with high alkaloid content). While we did not explicitly inquire about type of kratom consumed (e.g., whole leaf, extracts), 27% of participants cited consumption of kratom concentrates and/or extract shots when responding to the open-ended question about dose. Time for adverse effects to dissipate ranged from “instantly” to “one week.” Nearly one third of participants (30.6%) reported that adverse effects wore off in less than an hour, whereas 37.5% reported that effects wore off in 1–2 h and 6.9% in 3–4 h. A minority of participants (5.6%) shared that adverse effects took 9 h or greater to subside, and just one participant reported seeking out medical attention. For information pertaining to participants’ kratom use history and typical consumption patterns, we refer readers to our previously published manuscript [38].

### Themes

*N*= 69 participants provided a response to the open-ended question about contextual factors contributing to adverse reactions and four themes were generated from this data. *N*= 72 participants provided a response to the open-ended question about harm reduction strategies, of which five themes were generated. The percentages presented below use *N* = 74 as the denominator, representing the full group of participants who reported an adverse reaction.

**Table 2:** Themes

Themes	Codes
Contextual Factors	
Diet and Hydration Factors	- Not eating prior to kratom use or consuming on an empty stomach (39.2%) - Lack of hydration (9.5%) - Consuming certain foods with kratom (e.g., greasy or spicy foods) (8.1%)
Drug Interactions and Co-use With Other Substances	- Tobacco and nicotine products (12.2%) - Cannabis (9.5%) - Kava (6.8%) - Alcohol (4.1%) - Ibuprofen (1.4%) - Lexapro (escitalopram) (1.4%)
Set and Setting	- Mindset (e.g., anxiety) during or prior to use (2.7%) - Physical setting (e.g., overstimulating environment) during use (2.7%)
Kratom Use Topography	- Time of day consumed (2.7%) - Rate of consumption (1.4%) - Consumption of kratom concentrates (1.4%) - Low tolerance to kratom (1.4%)
**Harm Reduction Strategies Adopted in Attempt to Mitigate Adverse Reactions**	
Moderated Diet and Hydration	- Drinking water or another non-kratom beverage (52.7%) - Eating food (36.5%) - Vomiting (12.2%)
Rest and Recovery	- Sitting, lying down, or resting (9.5%) - Going to sleep (2.7%) - Waiting for effects to wear off (2.7%)
Moderated Use of Kratom and Other Substances	- Stopping kratom use or pacing consumption (8.1%) - Stopping or pacing use of other substances (6.8%)
Took Medication or co-used with Another Substance	- Cannabis (4.1%) - Nicotine (1.4%) - Antacids (2.7%) - Ibuprofen (1.4%) - Dramamine (dimenhydrinate) (1.4%) - Ginger (1.4%)
Moderated Set and Setting	- Changing environment or physical setting (5.4%) - Physical exercise (1.4%) - Meditation/breathing exercises (1.4%)

### Contextual factors

#### Theme 1: diet and hydration factors

The most common contextual factor reported by participants was not eating prior to kratom use or consuming kratom on an empty stomach (39.2%). Additionally, 9.5% of participants reported lack of hydration contributed to their adverse experience, while 8.1% reported that consuming certain foods with kratom (e.g., greasy, spicy) was a precipitating factor.

#### Theme 2: drug interactions and co-use with other substances

Participants also reported that consuming kratom with other substances or certain medications played a role in experiencing negative or unwanted effects. Drugs that were reported to have been consumed at the time of participants’ most recent adverse reaction to kratom were tobacco and nicotine products (12.2%), cannabis (9.5%), kava (6.8%), alcohol (4.1%), ibuprofen (1.4%), and Lexapro (escitalopram) (1.4%). Overall, 29.7% of participants who reported a prior adverse reaction to kratom shared that co-ingestion with other substances was a factor contributing to negative or unwanted effects.

#### Theme 3: set and setting

A minority of participants shared that their mindset (e.g., “anxiety”) during or prior to kratom use (2.7%) or their physical setting (e.g., “overstimulating environment”) during kratom use (2.7%) was a contextual factor that impacted adverse effects.

#### Theme 4: kratom use topography

Kratom use topography factors were also reported, encompassing time of day kratom was consumed (2.7%), rate at which kratom was consumed (1.4%), consumption of kratom concentrates (1.4%), and low tolerance to kratom (1.4%).

### Harm reduction strategies adopted in attempt to mitigate adverse reactions

#### Theme 1: moderated diet and hydration

In attempt to lessen negative or unwanted kratom effects, 52.7% of participants reported drinking water or another non-kratom beverage, 36.5% reported eating food, and 12.2% reported vomiting.

#### Theme 2: rest and recovery

Participants shared that sitting, lying down, or resting helped to mitigate adverse effects (9.5%). Going to sleep (2.7%) and simply waiting for effects to wear off (2.7%) was also reported (e.g., before driving).

#### Theme 3: moderated use of kratom and other substances

Stopping kratom use or pacing consumption (i.e., intentionally moderating the rate or quantity of kratom intake) was also reported (8.1%), along with stopping or pacing use of other substances (6.8%), such as cannabis.

#### Theme 4: took medication or co-used with another substance

Participants also reported using cannabis (4.1%) or nicotine (1.4%) in attempt to mitigate adverse effects, as well as consuming antacids (2.7%), ibuprofen (1.4%), Dramamine (dimenhydrinate) (1.4%), and ginger (1.4%).

#### Theme 5: moderated set and setting

Finally, some participants described changing their environment or physical setting (5.4%), while others reported engagement in physical exercise (1.4%) and meditation/breathing exercises (1.4%).

## Discussion

This exploratory qualitative substudy aimed to generate preliminary data on the contextual factors that may contribute to adverse reactions to kratom, as well as expand our understanding of what strategies are employed in attempt to reduce adverse reactions. Most participants in the current study reported at least one prior experience of negative or unwanted acute kratom effects, whereas prior work on prevalence of adverse reactions has been mixed. For example, one study found that three quarters of participants reported gastrointestinal symptoms attributed to kratom use [40], while other data suggest that only a minority of kratom consumers report experiencing adverse effects [41, 42]. However, most prior US-based surveys were foundational to understanding kratom use more broadly and thus focused less specifically on adverse reactions [10, 41]. Still, even in recent work utilizing ecological momentary assessment to study real-time kratom use, acute effects skewed largely positive or neutral, even though participants could respond across a range of desired and unwanted effects [11]. However, the sample was comprised of highly experienced kratom consumers, which may have impacted findings. While participants in our study reported frequent kratom use, over half had been using kratom at this frequency for less than 1 year [38] and none had been using at this frequency for over 3 years, indicating that our sample included individuals who were relatively early in their use. Participant demographic characteristics were similar to other US-based samples of people who use kratom (e.g., predominantly white, male) [43], however, our sample was somewhat younger (mean age = 22.34) in comparison to prior work showing that people who use kratom are typically between the ages of 25–44 [44].

While prior experience of adverse reactions was common in the present study, we note that the side effects reported by participants tended to be milder in nature (e.g., nausea, anxiety; see our previously published manuscript for an in-depth overview [38]). While more serious side effects have also been documented in the literature (e.g., toxicity, fatalities) [21], these types of events are typically rare [45]. However, given our smaller sample size and the fact that participants tended to be relatively early in their kratom use, more severe reactions may have been underrepresented in the present work.

### Contextual factors associated with adverse reactions to Kratom

Diet and hydration factors (e.g., not eating or drinking prior to kratom use) were by far the most frequently cited contextual factors associated with adverse reactions to kratom. This finding aligns with existing literature suggesting that gastrointestinal symptoms (e.g., nausea) are among the most commonly reported side effects of kratom use [22, 41, 46, 47]. Adverse reactions were reported to occur across a range of kratom doses and methods of consumption. However, emerging evidence indicating that effects of kratom may be dose-dependent underscores the critical need for further work in this area [22, 41, 48, 49].

In addition to the important role of dietary and hydration factors [12], these data suggest that combining kratom with other substances such as alcohol, nicotine and cannabis may increase risk of experiencing acute adverse effects, consistent with limited prior data on this topic which suggests that polydrug exposure is a risk factor associated with adverse reactions [20, 50]. Likewise, it is unclear if risk for adverse events, or their severity, differ based on the use of whole-leaf kratom products, compared to extracts [51]. Of growing concern, it is also unknown whether these distinctions exist with regard to novel semi-synthetic products derived from the metabolite of mitragynine (7-hydroxymitragynine), a mu opioid receptor agonist with high binding affinity. While scientists do not consider these products to be “kratom,” some consumers may not differentiate between them when providing self-reports [52].

### Harm reduction implications

These findings have immediate harm-reduction implications for public health messaging, given that there is little credible public-facing information available on how to safely use kratom [53]. In particular, there is a lack of clear messaging informing consumers and would-be consumers that they must differentiate between types of kratom (e.g., whole leaf vs. extract) and, increasingly, between kratom-derived products—similar to how people who use cannabis must differentiate between cannabis and cannabis-derived products of varying potency. Individuals may also be unaware of the potential risks of combining kratom with other psychoactive substances. It is worth noting that participants used several harm-reduction strategies in attempt to reduce unwanted acute effects of kratom, such as stopping or pacing their use of kratom and other substances, modulating hydration and food intake, and engaging in rest and recovery behaviors. While prior work on kratom harm reduction is limited, some additional strategies identified in the literature include adjusting amount of kratom consumed following an adverse experience, taking periodic breaks in use, limiting use of extracts, and rotating between various product formulations in attempt to prevent kratom tolerance [12, 16].

### Limitations and future directions

The present pilot study should be interpreted in light of several limitations. First, the retrospective and self-report nature of survey data introduces the potential for recall bias. We also did not ask participants to indicate whether they perceived the harm reduction strategies they reported as effective, and written responses to this open-ended item were often brief and lacking in detail. Future work would benefit from in-depth qualitative interviews or more structured survey items that assess strategies in greater detail and ask participants to report on perceived efficacy.

Additionally, the sample was lacking in demographic diversity and data was collected from only two ethnobotanical tea bars in Colorado, limiting the generalizability of findings. Future studies should replicate these findings in larger and more diverse samples from a wider range of kratom-serving establishments across the US. Our study also lacked standardized reporting of kratom dose, and we did not inquire about the type of product consumed (e.g., whole-leaf, extracts). While product type was reported by some participants in the open-ended item about kratom dose, future work should assess product type more explicitly. Furthermore, there is likely considerable variability in kratom dose and potency across various kratom preparations and the products served at ethnobotanical tea bars, limiting the interpretability of dose-related findings and underscoring the need for future studies to corroborate self-report data with laboratory analysis of products when possible.

While adverse effects of kratom are increasingly well-documented, research examining contextual factors and harm reduction strategies is limited. Our pilot study represents an initial step in characterizing the strategies people report using in attempt to mitigate potential kratom-related harms. We hope these findings can serve as preliminary groundwork to inform more rigorous qualitative and quantitative research, including the development of an instrument to measure and test the efficacy of kratom harm reduction strategies.

## Data Availability

Data and materials are available from the corresponding author upon reasonable request.

## Abbreviation

DSM-5: Diagnostic and Statistical Manual of Mental Disorders , fifth edition

## References

[1] Kamble SH, Berthold EC, Kanumuri SRR, King TI, Kuntz MA, León F, et al. Metabolism of Speciociliatine, an overlooked Kratom alkaloid for its potential Pharmacological effects. AAPS J. 2022;24. 10.1208/s12248-022-00736-8. 10.1208/s12248-022-00736-8 PMC9932950 35854066

[2] Hanapi NA, Chear NJY, Azizi J, Yusof SR. Kratom alkaloids: interactions with Enzymes, Receptors, and cellular barriers. Front Pharmacol. 2021. 10.3389/fphar.2021.751656. 34867362 10.3389/fphar.2021.751656 PMC8637859

[3] Obeng S, Leon F, Patel A, Zuarth Gonzalez JD, da Silva LC, Restrepo LF, et al. Interactive effects of m-Opioid and Adrenergic-a2 receptor agonists in rats: Pharmacological investigation of the primary Kratom alkaloid Mitragynine and its metabolite 7-Hydroxymitragynine. J Pharmacol Exp Ther. 2022;383. 10.1124/jpet.122.001192. 10.1124/jpet.122.001192 PMC9667981 36153006

[4] Obeng S, Kamble SH, Reeves ME, Restrepo LF, Patel A, Behnke M, et al. Investigation of the adrenergic and opioid binding Affinities, metabolic Stability, plasma protein binding Properties, and functional effects of selected Indole-Based Kratom alkaloids. J Med Chem. 2020;63. 10.1021/acs.jmedchem.9b01465. 10.1021/acs.jmedchem.9b01465 PMC7676998 31834797

[5] Mukhopadhyay S, Gupta S, Wilkerson JL, Sharma A, McMahon LR, McCurdy CR. Receptor selectivity and therapeutic potential of Kratom in substance use disorders. Curr Addict Rep. 2023. 10.1007/s40429-023-00472-9.

[6] Chen Y, McCurdy C, Mottinelli M, Canal C. Probing the activity of Mitragyna speciosa (Kratom) alkaloids at serotonin G Protein-Coupled receptors. FASEB J. 2022;36. 10.1096/fasebj.2022.36.s1.r2407.

[7] Grundmann O, Hendrickson RG, Greenberg MI. Kratom: History, pharmacology, current user trends, adverse health effects and potential benefits. Dis Mon. 2023. 10.1016/j.disamonth.2022.101442. 69. 35732553 10.1016/j.disamonth.2022.101442

[8] American Kratom Association. Natural Kratom Products vs. Adulterated Kratom Products—Where the FDA Went Wrong [Internet]. 2019 [cited 2024 Nov 17]. https://www.csakratom.org/uploads/AKA_Natural_vs_Adulterated_Kratom_Products_4c67264169.pdf?updated_at=2022-11-01T20:22:09.340Z. Accessed 17 Nov 2024.

[9] Grundmann O, Veltri CA, Morcos D, Knightes D, Smith KE, Singh D, et al. Exploring the self-reported motivations of Kratom (Mitragyna speciosa Korth.) use: a cross-sectional investigation. Am J Drug Alcohol Abuse. 2022. 10.1080/00952990.2022.2041026. 35389321 10.1080/00952990.2022.2041026

[10] Smith KE, Lawson T. Prevalence and motivations for Kratom use in a sample of substance users enrolled in a residential treatment program. Drug Alcohol Depend. 2017;180. 10.1016/j.drugalcdep.2017.08.034. 10.1016/j.drugalcdep.2017.08.034 28950240

[11] Smith KE, Panlilio LV, Feldman JD, Grundmann O, Dunn KE, McCurdy CR, et al. Ecological momentary assessment of Self-Reported Kratom Use, Effects, and motivations among US adults. JAMA Netw Open Am Med Association. 2024;7:E2353401. 10.1001/jamanetworkopen.2023.53401. 10.1001/jamanetworkopen.2023.53401 PMC10818224 38277146

[12] Smith KE, Feldman JD, Dunn KE, McCurdy CR, Weiss ST, Grundmann O, et al. Examining the Paradoxical effects of kratom: a narrative inquiry. Front Pharmacol. 2023;14. 10.3389/fphar.2023.1174139. 10.3389/fphar.2023.1174139 PMC10196254 37214465

[13] Feldman JD, Schriefer D, Smith KE, Weiss ST, Butera G, Dunn KE, et al. Omissions, Ambiguities, and underuse of causal assessment tools: a systematic review of case reports on patients who use Kratom. Curr Addict Rep. 2023. 10.1007/s40429-023-00466-7.37266191

[14] Alsarraf E, Myers J, Culbreth S, Fanikos J. Kratom from head to Toe—Case reviews of adverse events and toxicities. Curr Emerg Hosp Med Rep. 2019;7. 10.1007/s40138-019-00194-1.

[15] Hill K, Grundmann O, Smith KE, Stanciu CN. Prevalence of Kratom use disorder among Kratom consumers. J Addict Med. 2024;18. 10.1097/ADM.0000000000001290. 10.1097/ADM.0000000000001290 38441236

[16] Rogers JM, Colvin K, Epstein DH, Grundmann O, McCurdy CR, Smith KE. Growing pains with kratom: experiences discussed in subreddits contrast with satisfaction expressed in surveys. Front Pharmacol [Internet]. 2024;15. https://www.frontiersin.org/journals/pharmacology/articles/10.3389/fphar.2024.1412397 10.3389/fphar.2024.1412397 PMC11211595 38948457

[17] Smith KE, Epstein DH, Weiss ST. Controversies in Assessment, Diagnosis, and Treatment of Kratom Use Disorder. Curr Psychiatry Rep. Springer; 2024. pp. 487–96. 10.1007/s11920-024-01524-1 10.1007/s11920-024-01524-1 PMC11344726 39134892

[18] Swart BB, Reznikoff C, Steen K. Isolated Kratom use disorder treated with Extended-Release buprenorphine taper. J Addict Med [Internet]. 2024;18(5):602––604. https://journals.lww.com/journaladdictionmedicine/fulltext/2024/09000/isolated_kratom_use_disorder_treated_with.23.aspx 10.1097/ADM.0000000000001328 38776432

[19] American Psychiatric Association. Diagnostic and Statistical Manual of Mental Disorders, 5th Edition. Diagnostic and Statistical Manual of Mental Disorders, 5th Edition. 2013. 10.1176/appi.books.9780890425596.893619

[20] Corkery JM, Streete P, Claridge H, Goodair C, Papanti D, Orsolini L, et al. Characteristics of deaths associated with Kratom use. J Psychopharmacol. 2019;33. 10.1177/0269881119862530. 10.1177/0269881119862530 31429622

[21] Post S, Spiller HA, Chounthirath T, Smith GA. Kratom exposures reported to united States poison control centers: 2011–2017. Clin Toxicol. 2019;57. 10.1080/15563650.2019.1569236. 10.1080/15563650.2019.1569236 30786220

[22] Smith KE, Rogers JM, Dunn KE, Grundmann O, McCurdy CR, Schriefer D, et al. Searching for a signal: Self-Reported Kratom Dose-Effect relationships among a sample of US adults with regular Kratom use histories. Front Pharmacol. 2022;13. 10.3389/fphar.2022.765917. 10.3389/fphar.2022.765917 PMC8921773 35300296

[23] Martens MP, Ferrier AG, Sheehy MJ, Corbett K, Anderson DA, Simmons A. Development of the protective behavioral strategies survey. J Stud Alcohol. 2005;66. 10.15288/jsa.2005.66.698. 10.15288/jsa.2005.66.698 16329461

[24] Prince MA, Jenzer T, Brown W, Hetelekides EM, Mumm RA, Collins RL. Examining cannabis protective behavioral strategy use using multiple methods. Drugs Alcohol Today. 2019;19. 10.1108/DAT-10-2018-0061. 10.1108/dat-10-2018-0061 PMC8319839 34335859

[25] Piercey CJ, Gray B, Sung A, Henry D, Karoly HC. Protective behavioral strategies for psychedelic use: A mini review of the Evidence. psychedelic medicine. Mary Ann Liebert Inc; 2024. 10.1089/psymed.2023.0052. 10.1089/psymed.2023.0052 PMC11658379 40051484

[26] Pedersen ER, Huang W, Dvorak RD, Prince MA, Hummer JF, Anthenien AM, et al. The protective behavioral strategies for marijuana scale: further examination using item response theory. Psychol Addict Behav. 2017;31. 10.1037/adb0000271. 10.1037/adb0000271 PMC5546985 28703616

[27] Peterson R, Kramer MP, Pinto D, De Leon AN, Leary AV, Marin AA, et al. A comprehensive review of measures of protective behavioral strategies across various risk factors and associated PBS-related interventions. Exp Clin Psychopharmacol Am Psychol Association. 2021;29:236–50. 10.1037/pha0000498. 10.1037/pha0000498 34264735

[28] Fernández-Calderón F, Díaz-Batanero C, Barratt MJ, Palamar JJ. Harm reduction strategies related to dosing and their relation to harms among festival attendees who use multiple drugs. Drug Alcohol Rev. 2019;38. 10.1111/dar.12868. 10.1111/dar.12868 PMC6338512 30302851

[29] Vidal Giné C, Fernández Calderón F, López Guerrero J. Patterns of use, harm reduction strategies, and their relation to risk behavior and harm in recreational ketamine users. Am J Drug Alcohol Abuse. 2016;42. 10.3109/00952990.2016.1141211. 10.3109/00952990.2016.1141211 27052358

[30] Edwards D, Csontos J, Pascoe MJ, Westwell A, Gillen E, Bennett C et al. A rapid scoping review of harm reduction strategies for ecstasy (MDMA) users in recreational settings. 2022; 10.21203/rs.3.rs-2178425/v1

[31] Erinoso O, Daugherty R, Kirk MR, Harding RW, Etchart H, Reyes A, et al. Safety strategies and harm reduction for methamphetamine users in the era of Fentanyl contamination: a qualitative analysis. Int J Drug Policy Elsevier. 2024;128:104456. 10.1016/j.drugpo.2024.104456 PMC11590564 38761461

[32] Palamar JJ, Barratt MJ. Prevalence of reagent test-kit use and perceptions of purity among ecstasy users in an electronic dance music scene in new York City. Drug Alcohol Rev. 2019;38. 10.1111/dar.12882. 10.1111/dar.12882 PMC6338488 30575155

[33] Piercey CJ, Pince CL, Karoly HC. Highlighting variability in Fentanyl test strip instructions using thematic content analysis. Harm Reduct J Springer. 2025;22:110. 10.1186/s12954-025-01252-6 PMC12186409 40551121

[34] Hurlocker MC, Pearson MR. Opioid protective behavioral strategies scale (OPBSS): development and psychometric evaluation. Exp clin psychopharmacol. American Psychological Association; 2024. 10.1037/pha0000738 PMC11839172 39207397

[35] De Leon AN, Dvorak RD, Smallman R, Arthur K, Piercey C. Using counterfactual thinking theory to change alcohol protective behavioural strategy use intentions. Br J Health Psychol. 2022;27. 10.1111/bjhp.12535. 10.1111/bjhp.12535 34076329

[36] Mian MN, Altman BR, Low F, Earleywine M. Development of the Protective Strategies for Psychedelics Scale: A novel inventory to assess safety strategies in the context of psychedelics. Journal of Psychopharmacology. SAGE Publications Ltd; 2023; 10.1177/02698811231214060 10.1177/02698811231214060 PMC10851634 38050326

[37] Striley CW, Hoeflich CC, Viegas AT, Berkowitz LA, Matthews EG, Akin LP, et al. Health effects associated with Kratom (Mitragyna speciosa) and polysubstance use: A narrative review. Subst Abuse. 2022. 10.1177/11782218221095873. 35645563 10.1177/11782218221095873 PMC9130800

[38] Piercey CJ, Bunch J, Cameron J, Ahern R, Packwood I, Bruning C, et al. Kratom use among ethnobotanical tea bar patrons in colorado: subjective drug effects, adverse reactions, and perceived benefits of use. Drug Alcohol Depend Rep [Internet]. 2025;16:100361. 10.1016/j.dadr.2025.100361. 40823326 10.1016/j.dadr.2025.100361 PMC12355151

[39] Braun V, Clarke V. Using thematic analysis in psychology. Qual Res Psychol. 2006;3. 10.1191/1478088706qp063oa.

[40] Coe MA, Pillitteri JL, Sembower MA, Gerlach KK, Henningfield JE. Kratom as a substitute for opioids: results from an online survey. Drug Alcohol Depend. 2019;202. 10.1016/j.drugalcdep.2019.05.005. 10.1016/j.drugalcdep.2019.05.005 31284119

[41] Grundmann O. Patterns of Kratom use and health impact in the US—Results from an online survey. Drug Alcohol Depend. 2017;176. 10.1016/j.drugalcdep.2017.03.007. 10.1016/j.drugalcdep.2017.03.007 28521200

[42] Garcia-Romeu A, Cox DJ, Smith KE, Dunn KE, Griffiths RR. Kratom (Mitragyna speciosa): user demographics, use patterns, and implications for the opioid epidemic. Drug Alcohol Depend. 2020;208. 10.1016/j.drugalcdep.2020.107849. 10.1016/j.drugalcdep.2020.107849 PMC7423016 32029298

[43] Rogers JM, Smith KE, Strickland JC, Epstein DH. Kratom use in the US: both a regional phenomenon and a white Middle-Class phenomenon? Evidence from NSDUH 2019 and an online convenience sample. Front Pharmacol. 2021;12. 10.3389/fphar.2021.789075. 10.3389/fphar.2021.789075 PMC8721145 34987402

[44] Covvey JR, Vogel SM, Peckham AM, Evoy KE. Prevalence and characteristics of self-reported Kratom use in a representative US general population sample. J Addict Dis. 2020. 10.1080/10550887.2020.1788914. 32657217 10.1080/10550887.2020.1788914

[45] Vadiei N, Evoy KE, Grundmann O. The impact of diverse Kratom products on use Patterns, Dependence, and toxicity. Curr Psychiatry Rep. Springer;; 2025. pp. 1–9. 10.1007/s11920-025-01631-7 40762659

[46] Swogger MT, Hart E, Erowid F, Erowid E, Trabold N, Yee K, et al. Experiences of Kratom users: A qualitative analysis. J Psychoact Drugs. 2015;47. 10.1080/02791072.2015.1096434. 10.1080/02791072.2015.1096434 PMC12818578 26595229

[47] Singh D, Narayanan S, Vicknasingam B. Traditional and non-traditional uses of Mitragynine (Kratom): A survey of the literature. Brain Res Bull. 2016. 10.1016/j.brainresbull.2016.05.004. 27178014 10.1016/j.brainresbull.2016.05.004

[48] Singh D, Narayanan S, Grundmann O, Dzulkapli E, Bin VB. Effects of Kratom (Mitragyna speciosa Korth.) use in regular users. Subst Use Misuse. 2019;54. 10.1080/10826084.2019.1645178. 10.1080/10826084.2019.1645178 31347441

[49] Smith KE, Rogers JM, Schriefer D, Grundmann O. Therapeutic benefit with caveats? Analyzing social media data to understand the complexities of Kratom use. Drug Alcohol Depend. 2021;226. 10.1016/j.drugalcdep.2021.108879. 10.1016/j.drugalcdep.2021.108879 PMC8355181 34216869

[50] Kerrigan S, Basiliere S, Kratom. A systematic review of toxicological issues. WIREs Forensic Sci. 2022;4. 10.1002/wfs2.1420.

[51] Grundmann O, Garcia-Romeu A, McCurdy CR, Sharma A, Smith KE, Swogger MT, et al. Not all Kratom is equal: the important distinction between native leaf and extract products. Addiction. 2024;119. 10.1111/add.16366. 10.1111/add.16366 37814405

[52] Smith KE, Boyer EW, Grundmann O, McCurdy CR, Sharma A. The rise of novel, semi-synthetic 7-hydroxymitragnine products. Addiction. John Wiley and Sons Inc; 2024. 10.1111/add.16728 10.1111/add.16728 39627873

[53] Hill K, Gibson S, Grundmann O, Smith KE, Ballard J, Stanciu CN. Evaluating health information provided to Kratom consumers by good manufacturing practice-qualified vendors. Subst Abuse Treat Prev Policy BioMed Cent Ltd. 2023;18. 10.1186/s13011-023-00531-4. 10.1186/s13011-023-00531-4 PMC10088264 37041624

